# A 10-year longitudinal evaluation of science policy interventions to promote sex and gender in health research

**DOI:** 10.1186/s12961-021-00741-x

**Published:** 2021-06-15

**Authors:** Jenna Haverfield, Cara Tannenbaum

**Affiliations:** 1 Institute of Gender and Health, Canadian Institutes of Health Research, Montréal, QC Canada; 2 Faculté de Médecine et Pharmacie, Université de Montréal, Montréal, QC Canada

**Keywords:** Sex and gender, Science policy, Evaluation, Funding agency

## Abstract

**Background:**

Over the past decade, the Canadian Institutes of Health Research (CIHR) has implemented multicomponent interventions to increase the uptake of sex and gender in grant applications. Interventions included mandatory reporting on applicant forms, development of resources for applicants and evaluators, and grant review requirements. Here, we aim to inform science policy implementation by describing the 10-year outcomes and lessons learned from these interventions.

**Methods:**

This is a prospective longitudinal study. The population is all applicants across 15 investigator-initiated CIHR competitions from 2011 to 2019 and grant evaluators from 2018 to 2019. Quantitative data were derived from applicants’ and grant evaluators’ mandatory reporting of sex and gender integration in the grants management database. The application was the unit of analysis. Trends in sex and gender uptake in applications were plotted over time, stratified by research area. Univariate logistic regression was used to assess associations between the sex of the applicant and the uptake of sex and gender, and the latter with funding success. Qualitative review of the quality and appropriateness of evaluators’ comments informed the development of discipline-specific training to peer review committee members. Feedback was compiled from a subset of evaluators on the perceived usefulness of the educational materials using a brief questionnaire.

**Results:**

Since 2011, 39,390 applications were submitted. The proportion that reported integration of sex rose from 22 to 83%, and gender from 12 to 33%. Population health research applications paid the greatest attention to gender (82%). Across every competition, applications with female principal investigators were more likely to integrate sex (odds ratio [OR] 1.60, 95% confidence interval [CI] 1.50–1.63) and gender (OR 2.40, 95% CI 2.29–2.51) than those who identified as male. Since 2018, applications that scored highly for the integration of sex (OR 1.92, 95% CI 1.50–2.50) and gender (OR 2.53, 95% CI 1.83–3.50) were more likely to be funded. Qualitative observations revealed persistent conflation of the terms sex and gender. Eighty-six percent of evaluators appreciated the tailored discipline-specific coaching.

**Conclusions:**

A number of policy interventions improved sex and gender uptake in grant applications, with higher success rates observed over time for applications that integrated sex and gender. Other funders’ action plans around sex and gender integration may be informed from our experiences of the timing, type and targets of the different interventions, specifically those directed at evaluators.

**Supplementary Information:**

The online version contains supplementary material available at 10.1186/s12961-021-00741-x.

## Background

Paradigm shifts in the way science is conducted and ethically governed pose continual challenges for research funders tasked with the responsibility to set and ensure international standards of excellence. For instance, twentieth-century science followed a male-centred reductionist paradigm. Males served as the research subject default, with females consistently underrepresented at all stages of the research pipeline [1]. Basic and preclinical research was largely conducted in male cells, tissues and animals [2], and men comprised the majority of participants in clinical trials [3]. This historical male bias in science stemmed from erroneous assumptions that male and female cells were identical [4], that female animals increased experimental variability [5], and that no major differences existed between men and women outside of their reproductive organs [4]. As a result, persistent inattention to sex and gender in health research seriously compromised the discovery, quality, reproducibility and applicability of scientific evidence [6, 7]. Understanding how sex and gender interact, and how they intersect with other biological and sociocultural factors, is now viewed as being critical to drive scientific and therapeutic discovery, address health inequities and achieve social equality [7, 8]. Despite this, analyses of scientific literature across multiple disciplines indicate that many researchers are still not applying a sex and gender lens to their research programmes, or are not integrating these variables appropriately [9–11].

Research funders have a role to play in ensuring that the quality, relevance and impact of scientific research benefits people of all sexes, genders and other identity characteristics [7]. Over the past decade, funding agencies worldwide have adopted various sex- and gender-based analysis (SGBA) policies to ensure that research funded by their organization pays thoughtful attention to sex and gender at all stages of the research process, from the research design to data collection, analysis and reporting. These agencies include, but are not limited to, the Canadian Institutes of Health Research (CIHR) [12], United States National Institutes of Health, European Commission, Irish Research Council and German Research Foundation [13]. However, there has been considerable variation in the way health research funders have implemented their SGBA policies.

The main objective of this paper is to assess the impact of a multicomponent SGBA action plan that CIHR implemented over the past decade on applicant and grant evaluator behaviour towards sex and gender in research. We provide lessons learnt for other funding organizations to operationalize SGBA science policy interventions within their organizations.

## Methods

### Study design

This is a prospective longitudinal study using mixed methods to assess outcomes related to the 10-year rollout of a multicomponent SGBA policy intervention at CIHR.

### Population and setting

The population is all applicants to Canada’s national health research funding programme and its grant evaluators. Applicants for research funding are independent academic researchers at any career stage from various health research areas who submit a grant proposal. Grant evaluators are a subset of more mid-to-senior career researchers with experience in obtaining grants who possess the required expertise to assess the concept, feasibility, quality and potential impact of the proposed research.

The setting is Canada’s national health research investigator-initiated funding competition (Open Operating Grant Program and Project Grant competition). The competition provides operating funds to support all areas of health research that align with CIHR’s mandate. The competition is designed to capture ideas with the greatest potential for important advances in fundamental or applied health-related knowledge, the healthcare system, and/or health outcomes, by supporting projects with a specific purpose and a defined endpoint. Competition calls are held twice-yearly with some exceptions due to organizational changes.

### Interventions

**Fig. 1:**
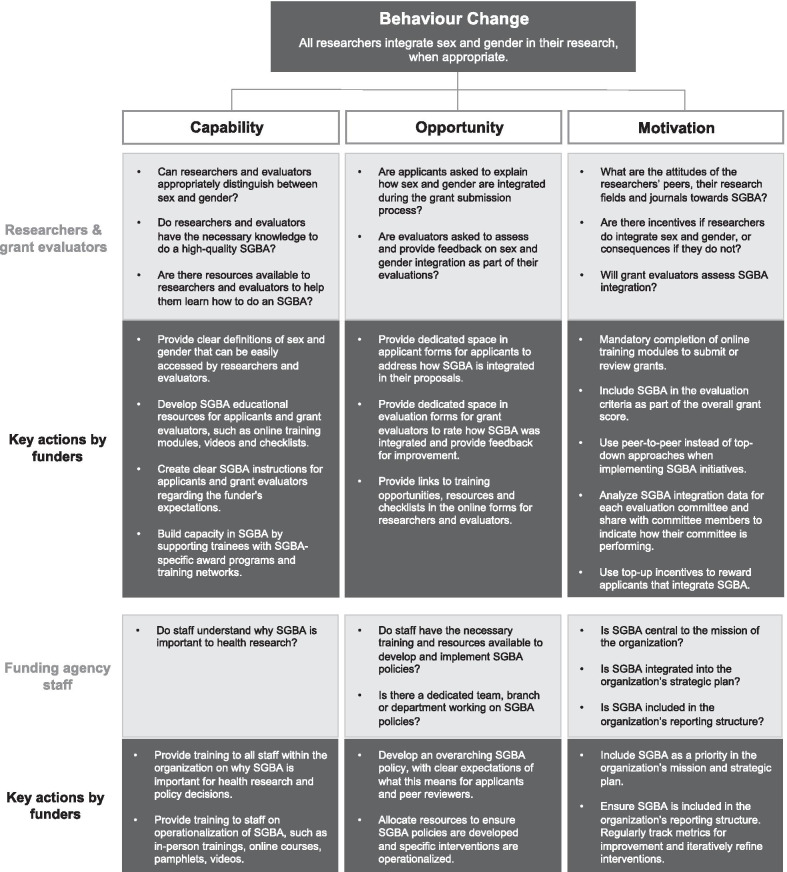
Application of a COM-B model for the successful development and implementation of sex- and gender-based analysis (SGBA) policies for research funders

**Fig. 2:**
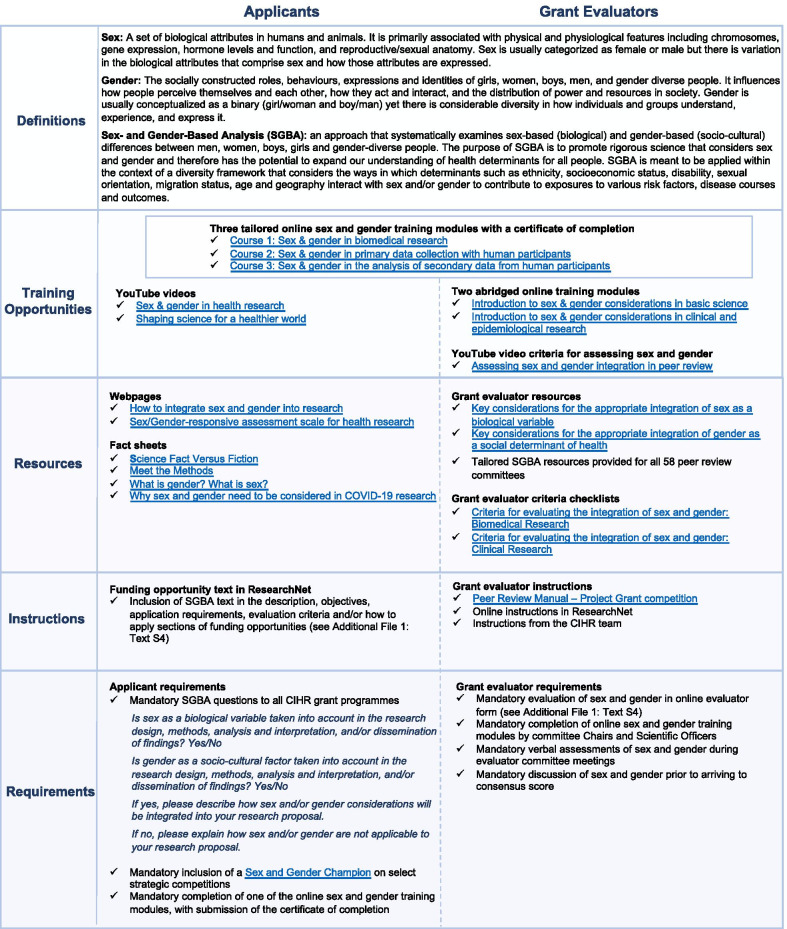
Overview of SGBA research policy interventions by a federal funding agency

Figures 1 and 2 describe a series of theory-informed SGBA interventions for applicants and evaluators that CIHR implemented in response to the Government of Canada’s Health Portfolio SGBA Policy. The SGBA policy, which came into effect in 2009, requires that SGBA be used “to develop, implement and evaluate the Health Portfolio’s research, legislation, policies, programs and services to address the different needs of women and men”. Intervention strategies that are driven by theoretical frameworks lead to more successful and sustainable behaviour change and help explain the success or failure of various interventions [14].

The multicomponent SGBA intervention reflects the capability, opportunity and motivation model of behaviour change (COM-B model) (Fig. 1). Michie et al. suggest that human behaviour is a function of the interaction between three key determinants: capability, opportunity and motivation [14]. When an individual has (a) the capability to engage in a particular action (e.g., the knowledge and skills of when and how to appropriately integrate sex and gender in research), (b) the opportunity to engage in that action (e.g., dedicated space on a grant proposal or evaluation form to explain and/or assess the appropriate inclusion or exclusion of sex and gender in the design, methods, analysis and reporting plan for the research), and (c) the motivation to engage in that action (e.g., mandatory peer review and/or peer pressure), then the action itself will occur. Essentially, this model of behaviour change recognizes that simply providing instruction is not enough to bring about a change in research culture or redefine scientific excellence. Instead, motivation and opportunities to show competence and appropriately engage in the new behaviour are also essential.

The SGBA interventions comprised five overarching components (Fig. 2): (1) education about definitions of sex and gender, (2) online and in-person training opportunities to persuade applicants and evaluators of the value and process for appropriately integrating sex and gender, (3) resources such as checklists and videos to enable the behaviour, (4) instructions to guide and prompt integration in grant proposals and assessments, and (5) requirements to ensure accountability such as mandatory reporting on applicant and evaluator forms or the inclusion in the team of a sex and gender champion for specific competitions. Interventions were targeted specifically for applicants, grant evaluators, or both. Several interventions were implemented for all of CIHR’s funding programmes, whereas others were specific to the investigator-initiated or strategic competitions. For instance, all funding programmes required applicants to indicate in an open-ended text box on the submission form how sex and/or gender were integrated in the proposal or justify their exclusion (Fig. 2 and Additional file 1: Text S2). The development and implementation of each intervention was an iterative process. The interventions were continually developed and modified in parallel with data analyses to inform next steps.

### Data collection and analysis

#### Quantitative data

Data were analysed from the grants management database across 15 investigator-initiated competitions between 2011 and 2019. The grants management database collects information submitted by applicants and grant evaluators in their online accounts, including their area of research and whether they identify as female or male. Since 2011, applicants are required to respond to two mandatory checkbox questions on whether sex and/or gender are integrated into their proposals (yes, no) and to justify its exclusion (Fig. 2 and Additional file 1: Text S2). Since 2018, grant evaluators must complete a checkbox rating the quality of integration of sex and gender as a strength, weakness, or not applicable for each submitted application. Evaluator data were available from three competitions in 2018 and 2019 (Additional file 1: Text S4).

The unit of analysis was the grant application. Every application submitted to the investigator-initiated competitions between 2011 and 2019 was included in the analyses, not just those that were funded, and no applications were excluded. To assess trends in sex and gender uptake in grant applications over time, the proportion of proposals that endorsed “‘yes” to CIHR’s mandatory questions on sex and gender integration was calculated [15]. Proportions were calculated for sex and gender integration separately for each competition over the 10-year period. A test for linear trend over time was calculated for sex and gender separately using a multiple-comparisons one-way analysis of variance (ANOVA) test (*p* < 0.05). To analyse trends across different research areas, data was stratified by one of four research areas, as declared by the applicant: (1) biomedical, (2) clinical, (3), health systems services and (4) population health [16]. To determine if sex and gender integration differed between female and male applicants, we used univariate logistic regression analysis with sex of the applicant as the independent variable. Analyses were not adjusted for age, career stage or other identity characteristics as data were not available for all competitions.

To investigate whether the quality of sex and gender integration was associated with funding outcomes, we used evaluators’ assessments of the quality of sex and gender integration in each application they reviewed. Each application was evaluated by three independent evaluators. Applications that were rated as a “strength” by two or more evaluators were categorized as having high-quality integration, and those that were rated as a “weakness” by two or more evaluators were categorized as having weak integration. Applications that were rated as “not applicable” by two or more evaluators were not included in the analysis. Univariate logistic regression analyses assessed whether funding success (yes, no) was associated with the strength or weakness of integration. The results of the logistic regression analyses are reported as odds ratios (OR) with 95% confidence intervals (CI). All data were analysed using the Statistical Package for the Social Sciences, version 26 (SPSS; IBM Corp., Armonk, NY, USA) and GraphPad Prism, version 9 (San Diego, CA, USA).

To determine whether grant evaluators valued the launch of committee-specific SGBA resources tailored to their specific committee mandate during the Fall 2019 competition, feedback was obtained from evaluators in the form of an online self-administered questionnaire. Grant evaluators that received committee-specific SGBA resources during the Fall 2019 competition (*n* = 624) were emailed a link to the questionnaire shortly after their committee meetings. The online form queried whether the materials deepened understanding of sex and gender integration and helped their review. Evaluators were given approximately 2 weeks to complete the survey. A total of 136 responses were received (response rate = 22%), and a tabulation of the multiple-choice questions was performed.

#### Qualitative data

Since 2018, evaluators were asked to provide a rationale for their rating in an open-ended text box on the evaluation form, with recommendations for improvement to the applicant (Fig. 2 and Additional file 1: Text S4). To gain insight into how grant evaluators were approaching their SGBA reviews, a random sampling approach across all four research pillars was used. Grant evaluator open-text comments from 5% of grants (*n* = 141 applications and *n* = 393 total reviews [3 reviews per application]) submitted to the Spring 2018 Project Grant competition were studied and explored for quality and appropriateness. We assessed quality as a function of whether thoughtful criticisms and recommendations for improvement were proposed, and appropriateness as instances where the terms sex and gender were used correctly or incorrectly.

## Results

### Researchers are paying more attention to sex and gender

**Fig. 3:**
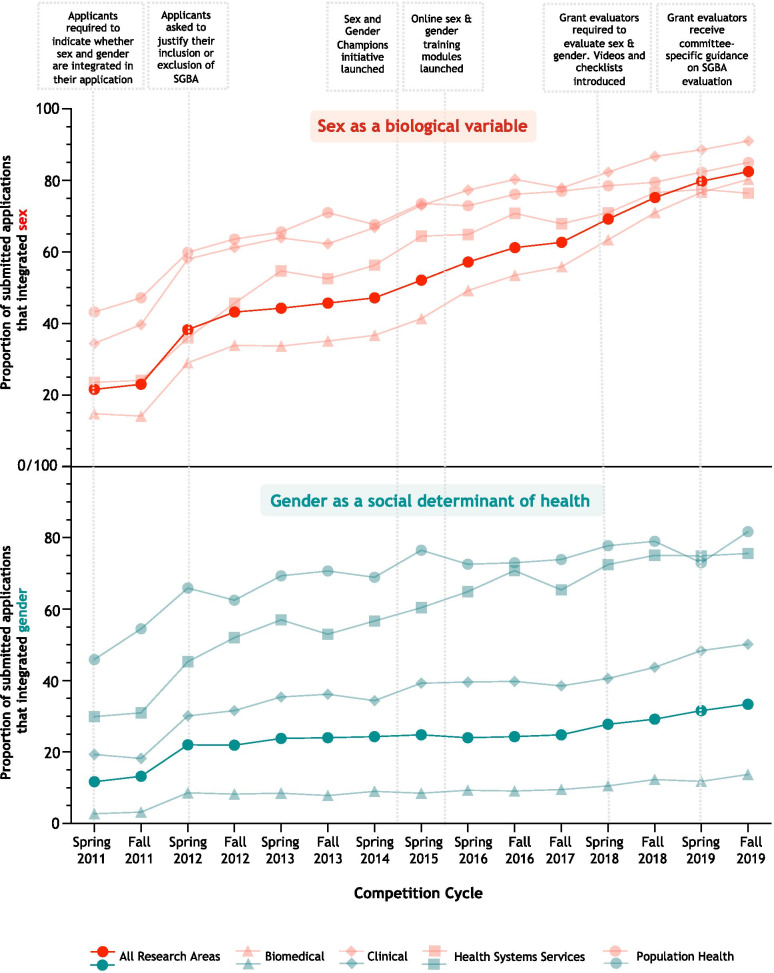
Integration of sex and gender in grant proposals submitted to CIHR’s largest investigator-initiated competition from 2011 to 2019. Data represent the proportion of applications submitted to each investigator-initiated competition cycle between 2011 and 2019 that accounted for sex and gender, disaggregated by research area. Dotted overlays highlight key SGBA interventions implemented by CIHR at several time points throughout the 10-year period

Analysis of data collected from 39,390 applications submitted to CIHR’s investigator-initiated programme across 15 competitions over the past 10 years (median number of applications submitted for each competition; *n* = 2,484; Min: *n* = 2183; Max: *n* = 3813) revealed a significant trend in the proportion of applicants reporting that sex and gender was integrated in their research proposals (*p* value for linear trend for sex and gender = *p* < 0.0001). The integration of sex in grant proposals was more pronounced, with an overall 3.8-fold increase, from 22% of applications in 2011 to 83% in 2019 (Fig. 3). The proportion of proposals accounting for gender increased 2.8-fold, from 12% of applications in 2011 to 33% in 2019. Figure 3 illustrates the timing of each of the various components of the SGBA intervention in relation to trends in sex and gender integration.

Sex and gender integration improved across all research areas; however, the extent varied by research area (Fig. 3). Biomedical research exhibited the greatest uptake in sex integration over the 10-year period, increasing fivefold from 15% of applications in 2011 to 80% in 2019. Clinical research had the highest proportion of applications integrating sex in the most recent competition analysed, with 91% of applications submitted to the Fall 2019 competition accounting for sex. Health systems services and population health grant proposals displayed the greatest integration of gender, with up to 82% of applications submitted to the Fall 2019 Project Grant competition accounting for gender. As expected, the proportion of biomedical applications integrating gender was low across all competitions. The majority of research in this area uses in vitro model systems, cell lines and animal models, and gender is usually not relevant.

### Female applicants are more likely to integrate sex and gender

**Fig. 4:**
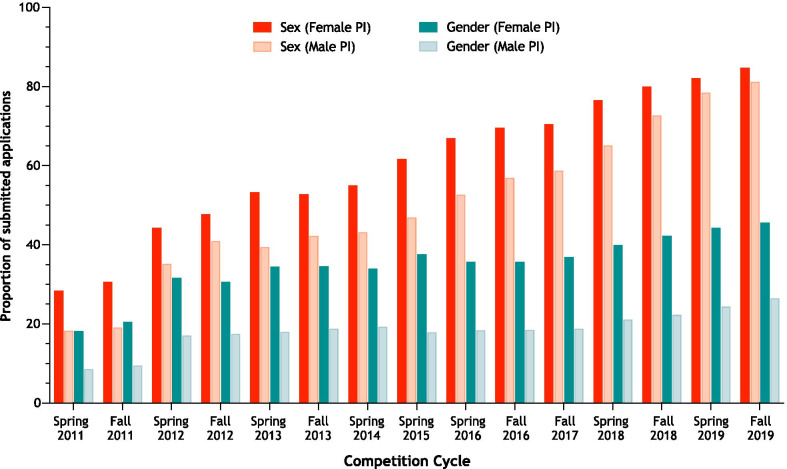
Trends in sex and gender integration by male and female applicants. Data represent the proportion of applications submitted to each investigator-initiated competition cycle between 2011 and 2019 that accounted for sex and gender, disaggregated by principal investigator sex

On average, the proportion of applications submitted by female investigators for each competition was 34%. Across each competition over the 10-year period, applications submitted by female compared with male investigators were more likely to integrate sex (OR 1.60, 95% CI 1.50–1.63) and gender (OR 2.40, 95% CI 2.29–2.51) (Fig. 4).

### Integrating sex and gender is associated with funding success

**Fig. 5:**
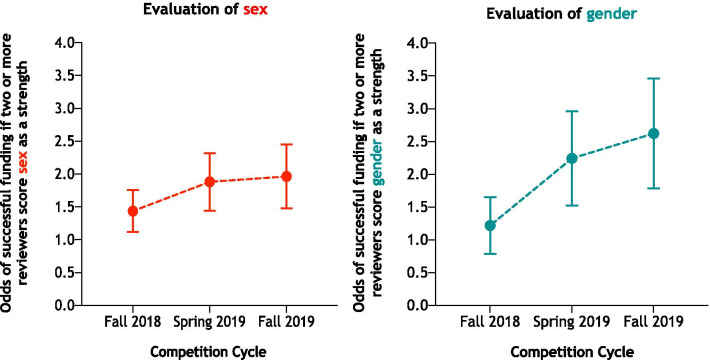
Association between integration of sex and gender in grant submissions and successful funding outcomes. Data represent the odds ratios and 95% confidence intervals of successful funding if two or more grant evaluators score sex or gender as a strength across three competition cycles between 2018 and 2019

The average success rate of applications submitted to all competitions over the 10-year period was 16% (Min = 13%; Max = 18%). Analysis of *n* = 21,336 individual grant evaluator scores for SGBA across *n* = 7 112 independent applications in 2018–2019 revealed that proposals rated highly for their integration of sex by two or more grant evaluators were more likely to be funded (Fall 2018: 1.41, 95% CI 1.13–1.77; Spring 2019: OR 1.85, 95% CI 1.46–2.34; Fall 2019: OR 1.92, 95% CI 1.50–2.50) (Fig. 5). Proposals that were rated highly for their integration of gender by two or more grant evaluators were more likely to be funded in the latter two competitions (Fig. 5) (Spring 2019: OR 2.17, 95% CI 1.56–3.00; Fall 2019: OR 2.53, 95% CI 1.83–3.50).

### Mandating evaluator feedback requires coaching

Assessment of grant evaluator feedback in the open-ended text boxes in the Spring 2018 competition revealed that one in four evaluators failed to provide substantive comments. Almost half of evaluators pasted or reiterated text from the application, rather than providing critical comments or recommending improvements in the way SGBA was considered in the research proposed. Evaluators for biomedical research most often indicated that SGBA was not applicable to the research. Evaluators for biomedical and clinical research were less likely to provide a quality review than evaluators reviewing health services and population health grants. There were cases across all pillars where grant evaluators and/or applicants inappropriately used or conflated the terms sex and gender.

After the introduction of tailored SGBA support material for each of CIHR’s 58 peer review committees [17], 86% of evaluators indicated appreciation for receiving tailored coaching by discipline. Sixty-nine percent reported that the educational materials deepened their understanding of how sex and gender are relevant to research within their committee mandate. Seventy-six percent believed the materials and discussions around discipline-specific examples were helpful and would contribute to a more rigorous assessment of the appropriate integration of sex and/or gender in applications reviewed at future meetings.

## Discussion

A series of science policy interventions implemented by a national funding agency not only advanced the uptake of sex and gender considerations into research content by applicants, but over time, successfully shifted the culture of research assessment to include sex and gender as an essential metric of scientific excellence. A theory-driven approach to organizational behavioural change—comprising building capacity, providing the opportunity to engage, and gradually escalating the motivation to appropriately integrate sex and gender—was coupled with regular data measurement to review and improve on each intervention. We offer important lessons for science policy implementation by interpreting the inflection points in sex and gender uptake as a function of the timing, type and targets of the policy interventions that were trialled.

First, if the timing of new science policies aligns with news media trends or current events, there may be greater uptake. In the case of Canada’s Health Portfolio’s new SGBA policy in 2009, the introduction of the policy occurred in a void, after a routine government audit report suggested that Canada’s commitment to gender-based analysis was not being adequately enforced. As such, the policy resulted from an internal government-driven directive that may have lacked academic visibility. Following this directive, CIHR made a commitment to improve the quality of science and took leadership to roll out a series of iterative multicomponent SGBA interventions. Clear definitions for sex and gender (Additional file 1: Text S1) were published on the CIHR website, and CIHR required applicants to report whether sex or gender was integrated in their proposals during the grant submission process (Additional file 1: Text S2). When the majority of applicants first responded in the negative, CIHR motivated applicants to reflect further by asking applicants to justify their choice of omission. This latter intervention yielded the first significant inflection point in the uptake of sex and gender considerations, which then plateaued for several years. A take-home message is that checkbox accountability for considering sex and gender may be more effective when negative flags require written justification from applicants.

Second, interactive capacity-building, dialogue and support beyond mere instruction are needed to foster behavioural change. An analysis of the applicants’ justifications for omitting sex and gender, published in 2014, revealed poor understanding of when and how to integrate sex and gender in research [15]. In order to build greater capacity for sex and gender integration, CIHR subsequently launched two educational program: the sex and gender champion programme in 2014, and online training in 2015. The sex and gender champion provided leadership and expertise for each research team to integrate sex and gender [18]. However, there were far too few researchers in Canada who qualified as sex and gender champions to require their integration for all research teams in the investigator-initiated programme, so the requirement could only be applied to specific strategic competitions. Although the inclusion of a sex and gender champion never became a requirement for applications to CIHR’s investigator-initiated competition, this programme may have had an indirect effect on the number of applications accounting for sex and gender as there is some overlap between those who apply for both strategic and investigator-initiated competitions. On the negative side, we heard that some champions were only invited to participate in the team at the last minute, so could not meaningfully contribute to the grant proposal. More effective methods to foster dialogue around sex and gender integration may result from alternate triggers and checkpoints, either through peer-to-peer follow-up conversations when evaluators provide criticism to applicants for weak integration, or amongst evaluators during peer review.

The development of interactive online training modules led to an increase in the integration of sex and gender integration through 2016 and 2017 [19]. These free courses, which educate applicants and reviewers on why sex and gender is important for health research and identifies methods for conducting sex and gender analyses in various health research contexts, have been shown to improve knowledge, self-efficacy and the intent to appropriately integrate sex and gender in research [20]. The online training modules require a pass grade on a post-test to receive a certificate of completion. Submission of the certificate of completion of at least one of the three online courses is a requirement for applicants to many of CIHR’s competitions and is recommended for applicants and grant evaluators of the investigator-initiated competition.

Third, targeting applicants alone to adopt new science policies without concomitant pressure by evaluators to ensure quality uptake may not be effective. Since 2018, there has been an upward trend in sex and gender integration, which corresponded with a new requirement for grant evaluators to factor the quality of sex and gender integration into the overall grant score. As with applicants, evaluators needed coaching and targeted tools to better understand and apply the criteria for appropriate integration. Material was developed and tailored specifically to each committee mandate to address why sex and gender is relevant to their committee, illustrate committee-specific SGBA data trends and explain what is expected of grant evaluators regarding SGBA. In-person discussions with committee Chairs, instructions for the Chairs and Scientific Officers of the granting panels to complete the training modules, and peer pressure to provide quality assessments during evaluation discussions finally moved the needle in a meaningful fashion. More intense interventions for evaluators culminated in a robust effect of sex and gender integration being associated with funding success, and hint at a sustainable change in culture. Now that favourable reviews of sex and gender in submitted proposals are associated with funding success, applicants will be more motivated to provide rigorous methodological justification in their proposals.

Fourth, management of science policies is not possible without measuring impact. CIHR annually tracks and reports the proportion of its research applications that incorporate sex and gender to government, in line with the Health Portfolio SGBA policy. Regular assessments prompt reflection on the effectiveness of different interventions and permit improvements via future performance planning. The finding that sex and gender integration increased over time and was ultimately associated with funding success likely indicates the proposals grew stronger as a result of the clear message from the funding agency that research that integrates sex and gender is valued. When grant evaluators are required to review the sex and gender integration as part of their assessment of scientific excellence, it influences the overall grant score and in turn results in higher-quality science.

The health research funding context in Canada may be different than in other countries, where funders may not be held accountable to an SGBA Health Portfolio policy. Despite the Canadian context, several of our findings have been confirmed by other funders [11, 21, 22]. The terms sex and gender are routinely conflated by both applicants and grant evaluators, and in many cases, additional education is needed about what constitutes appropriate integration and why it is important. Furthermore, the finding that female compared to male investigators are more likely to account for sex and gender across all of CIHR’s research areas is consistent with other reports that female principal investigators prioritize sex and gender in their research programmes [15, 23, 24]. Recent gender equity policies introduced by CIHR to mitigate the effects of the coronavirus disease 2019 (COVID-19) pandemic on female investigators not only increased the number of applications from female investigators and, consequently, their proportion of grants funded, but was also associated with improved integration of sex and gender in the content of the COVID-19 grant proposals [25].

A limitation of this longitudinal analysis is that causality cannot be attributed to any one intervention component in isolation. The uptake of sex and gender in grant proposals reflects the cumulative impact of a series of complex strategies rolled out over a 10-year period that ultimately shifted the culture of the organization. Increasing attention to sex and gender in research content is likely a result of multiple factors, including broader societal trends in the past 5 years and a shifting understanding of what constitutes scientific excellence. Additionally, other funders, as well as journals and universities, have recently increased requirements about sex and gender integration. Further limitations include the fact that gender is not relevant to most areas of basic science, so it is difficult to gauge an appropriate or target level of integration for the organization. Moreover, we were unable to determine associations between sex and gender uptake and other applicant other identity characteristics, such as age and career stage, due to a lack of available data. For the logistic regression analyses, we defined quality integration of sex and gender as a strong rating by two out of three evaluators, as SGBA methods are in constant flux, and evaluators are not always in agreement around SGBA “best practices”. Only 22% of the evaluators responded to the optional feedback questionnaire, potentially introducing bias to the results. Evaluators who did not answer the questionnaire may not have found value in the discipline-specific training materials.

## Conclusion

CIHR implemented a data-driven multicomponent intervention over a 10-year period to integrate sex and gender into grant proposals and thereby improve the quality, reproducibility and applicability of Canadian research. Sex and gender uptake increased over time and was eventually linked with grant success. The roll out of the SGBA policy interventions was an iterative process, with the timing, type and targets of the interventions requiring thoughtful reflection. To motivate applicants, funders may want to consider supporting evaluators first. Structural changes to the submission and evaluation forms were helpful for triggering and measuring uptake, but the quality of the sex and gender content may suffer if insufficient attention is given to building sex and gender competency among applicants and reviewers. Tracking quantifiable metrics in the absence of quality assessment is, therefore, not enough. Offering online resources is a key part of training. One-to-one coaching and peer-to-peer dialogue tailored to discipline seem to enable sustainable culture change as it is challenging to develop blanket resources that address the diverse needs of the entire research community. We recommend implementing uniform changes across all investigator-initiated competitions rather than for strategic competitions only. Emphasis on the future integration of additional identity factors such as age and race is the next step, as well as intersections between these factors in order to ensure that research benefits everyone.

## Supplementary Information

**Additional file 1: Text S1.** CIHR’s definitions for sex, gender and SGBA.** Text S2.** Mandatory sex and gender questions for all CIHR competitions. **Text S3. **How SGBA is integrated into CIHR funding opportunities. **Text S4. **Evaluator instructions and evaluation requirements.

## Data Availability

Data are held in a repository at CIHR, within their mandate as a national funding agency. Data are confidential due to Canadian privacy legislation, and permission for analysis must follow the Canadian Tri-Council Policy Statement 2: Ethical Conduct for Research Involving Humans (available: https://ethics.gc.ca/eng/policy-politique_tcps2-eptc2_2018.html, accessed July 13, 2020). Researchers interested in addressing research questions related to the data used in this paper and other grant funding may contact the CIHR at Funding-Analytics@cihr-irsc.gc.ca.

## Abbreviations

CIHR: Canadian Institutes of Health Research
IGH: Institute of Gender and Health
OR: Odds ratio
CI: Confidence interval
SGBA: Sex- and gender-based analysis
COM-B model: Capability, opportunity and motivation model of behaviour change
