# Supplementary material for: A 10-year longitudinal evaluation of science policy interventions to promote sex and gender in health research
Source: Health Res Policy Syst. 2021 Jun 15;19:94. doi: 10.1186/s12961-021-00741-x (PMC8205200; doi:10.1186/s12961-021-00741-x)
Supplement: Supplementary file 1 — Additional file 1: Text S1. CIHR’s definitions for sex, gender and SGBA. Text S2. Mandatory sex and gender questions for all CIHR competitions. Text S3. How SGBA is integrated into CIHR funding opportunities. Text S4. Evaluator instructions and evaluation requirements. [file 12961_2021_741_MOESM1_ESM.docx]

**Additional file 1**

**Text S1: CIHR’s definitions for sex, gender and SGBA**

**Sex:** A set of biological attributes in humans and animals. It is primarily associated with physical and physiological features including chromosomes, gene expression, hormone levels and function, and reproductive/sexual anatomy. Sex is usually categorized as female or male but there is variation in the biological attributes that comprise sex and how those attributes are expressed.

**Gender:** The socially constructed roles, behaviours, expressions and identities of girls, women, boys, men, and gender diverse people. It influences how people perceive themselves and each other, how they act and interact, and the distribution of power and resources in society. Gender is usually conceptualized as a binary (girl/woman and boy/man) yet there is considerable diversity in how individuals and groups understand, experience, and express it.

**Sex- and Gender-Based Analysis (SGBA)**: an approach that systematically examines sex-based (biological) and gender-based (socio-cultural) differences between men, women, boys, girls and gender-diverse people. The purpose of SGBA is to promote rigorous science that considers sex and gender and therefore has the potential to expand our understanding of health determinants for all people. SGBA is meant to be applied within the context of a diversity framework that considers the ways in which determinants such as ethnicity, socioeconomic status, disability, sexual orientation, migration status, age and geography interact with sex and/or gender to contribute to exposures to various risk factors, disease courses and outcomes. Applying SGBA brings these considerations into focus and can help formulate health research, policies and programs that are relevant to the diversity of the Canadian population.

**Text S2: Mandatory sex and gender questions for all CIHR competitions**

For all CIHR funding opportunities, Nominated Principal Applicants (NPA) are asked three questions on ResearchNet. When the NPA clicks on the ‘sex’ or ‘gender’ hyperlink, or the green question mark, it takes them to the ['How to integrate sex and gender into research’](http://www.cihr-irsc.gc.ca/e/50836.html) resource page on the CIHR website. Even if sex and/or gender is not relevant, applicants are expected to justify why in the text box.


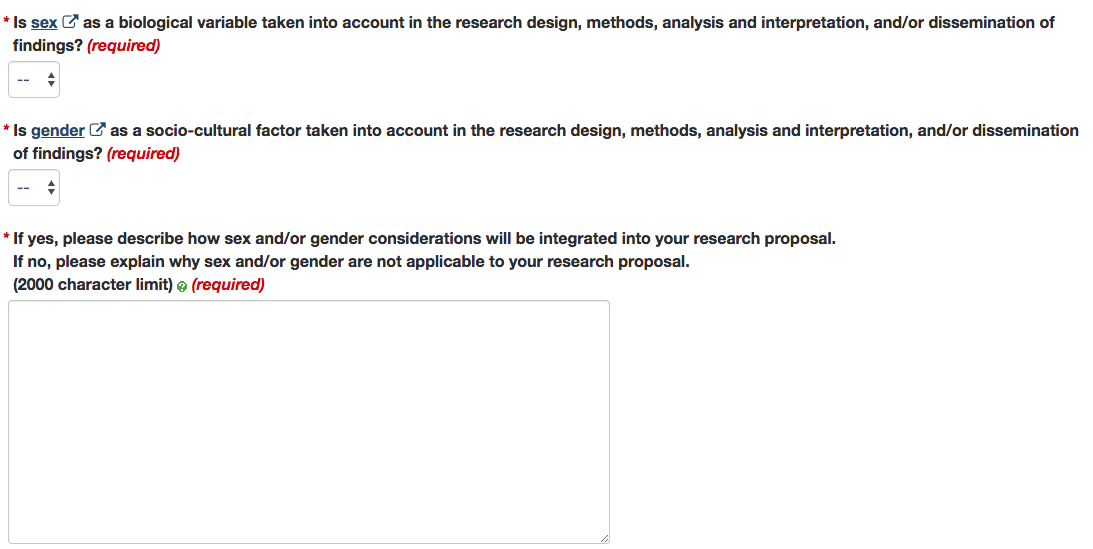


**Text S3: How SGBA is integrated into CIHR funding opportunities**

| **Funding Opportunity Section** | **Text Used** |
| --- | --- |
| **Description** | CIHR requires applicants to consider the integration of sex, as a biological variable, and gender, as a socio-cultural determinant of health, in their research where appropriate. Applicants should also consider other intersecting identity factors, such as age, race, ethnicity, culture, religion, geography, education, disability, income and sexual orientation where appropriate. CIHR’s position on sex and gender in health research is available on the [Sex, Gender and Health Research](http://www.cihr-irsc.gc.ca/e/50833.html) webpage. |
| **Application Requirements** | - Proposals must outline how sex, as a biological variable, gender, as a socio-cultural determinant of health, and other intersecting identity factors such as age, race, ethnicity, culture, religion, geography, education, disability, income and sexual orientation will be accounted for in the research design, methods, analysis, interpretation, and/or dissemination of findings, where appropriate. Applicants are encouraged to review the “[How to integrate sex and gender in research](http://www.cihr-irsc.gc.ca/e/50836.html)” section on the CIHR website. - The Nominated Principal Applicant/Principal Applicants/Co-Applicants must have successfully completed one of the three sex- and gender-based analysis [training modules](https://www.cihr-irsc-igh-isfh.ca/) available online through the CIHR Institute of Gender and Health and have submitted a Certificate of Completion (see How to Apply section). Please select and complete the training module most relevant to your research project. |
|  | ***Additional option**   - The team must include a [Sex and Gender Champion](http://www.cihr-irsc.gc.ca/e/50652.html) listed as a Nominated Principal Applicant or Principal Applicant. The Champion will be a researcher who possesses or acquires expertise in sex as a biological variable, or gender as a social determinant of health. |
| **Evaluation Criteria** | **Approach.** Quality and appropriateness of the [sex- and gender-based analysis+](http://www.cihr-irsc.gc.ca/e/50836.html) (SGBA+). SGBA+ refers to the consideration and examination of sex, gender and other identity factors (e.g., age, race, ethnicity, culture, religion, geography, education, disability, income and sexual orientation) at all stages of the research process including planning and implementation of the research project and related activities  ***Additional option**  **Team**  Qualifications and experience of the applicants in the proposed area of research, including the role of the Sex and Gender Champion. |
| **How to Apply** | **Upload the following under “Other”:**   - Certificate of Completion for the sex- and gender-based analysis training modules for (insert applicants required).   - After completing a training module, you will receive a PDF Certificate of Completion. Download this Certificate and upload the PDF here. Note that this document is mandatory (see Eligibility). |

**Text S4: Evaluator instructions and evaluation requirements**

For strategic competitions, evaluation instructions and requirements vary between funding opportunities, but for the CIHR Project Grant competition, the following instructions are provided in the [CIHR Peer Review Manual for Project Grants](http://www.cihr-irsc.gc.ca/e/49564.html):

“CIHR expects that all applicants will integrate sex and gender into their research designs when appropriate. Sex- and gender-based analysis (SGBA) is an approach that systematically examines sex-based (biological) and gender-based (socio-cultural) differences between men, women, boys, girls and gender-diverse people. The purpose of SGBA is to promote rigorous science that is sensitive to sex and gender and therefore has the potential to expand our understanding of health determinants for all people. Reviewers are asked to explicitly assess whether the integration of sex (as a biological variable) and/or gender (as a socio-cultural factor) is a strength, a weakness or not applicable to the proposal. Reviewers are also asked to comment on their assessment and to provide recommendations to the applicants on how they might improve the strength of their applications with respect to the integration of sex and/or gender. The [SGBA section of the CIHR website](http://www.cihr-irsc.gc.ca/e/32019.html) provides helpful resources for applicants and peer reviewers alike, including CIHR's definitions for sex, gender and SGBA, as well as information on applying SGBA to the development and assessment of research proposals.”

A screenshot of the peer review assessment page for SGBA on ResearchNet is provided below. It is a 2-step process, evaluators first select either Strength, Weakness or N/A, and then explain the rationale and writing recommendations for improvement in the text box.


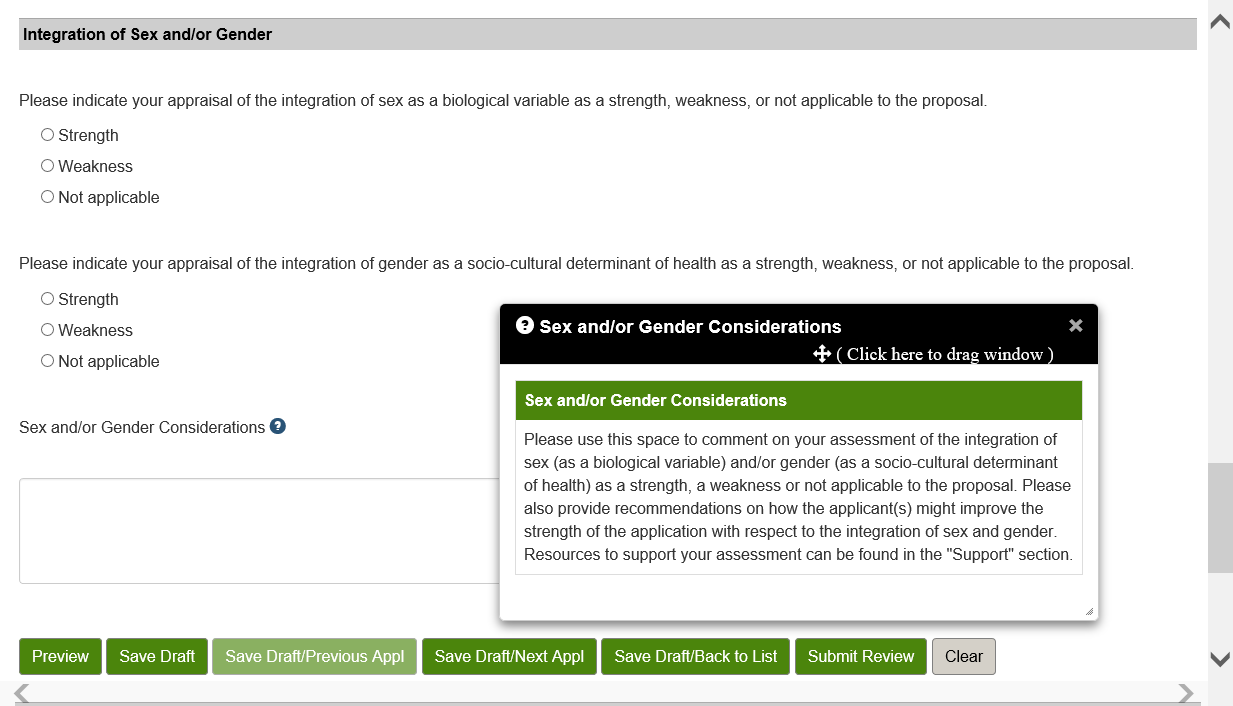


For CIHR’s recent COVID-19 competitions, the inclusion of sex and gender in grant evaluation criteria was expanded to include sex- and gender-based analyses **plus** (SGBA**+**).

The following wording was used in the evaluation criteria of CIHR’s second COVID-19 rapid response funding opportunity, which was also provided in the instructions to evaluators:

“Quality and appropriateness of the [sex- and gender-based analysis+](http://www.cihr-irsc.gc.ca/e/50836.html) (SGBA+). SGBA+ refers to the consideration and examination of sex, gender and other identity factors (e.g., age, race, ethnicity, culture, religion, geography, education, disability, income and sexual orientation) at all stages of the research process including planning and implementation of the research project and related activities – please visit [How to integrate sex and gender into research](http://www.cihr-irsc.gc.ca/e/50836.html) and [Why Sex and Gender Need to be Considered in COVID-19 Research](https://www.researchnet-recherchenet.ca/rnr16/vwOpprtntyDtls.do?prog=3309&view=currentOpps&org=CIHR&type=EXACT&resultCount=25&sort=program&all=1&masterList=true#moreinformation) for additional information”.
